# Treatment of Intussusception

**Published:** 1885-06-20

**Authors:** Frederick Treves

**Affiliations:** Surgeon to and Lecturer on Anatomy at the London Hospital


### THE TREATMENT OF INTUSSUSCEPTION.

By FREDERICK TREVES, F.R.C.S.,
Surgeon to and Lecturer on Anatomy at the London Hospital.

Jan. 24, 1885.—The treatment of intussusception should be prompt and active, and no reliance is to be placed upon expectant measures. In dealing with the detailed treatment of intussusception, it will be most convenient to limit the matter to the treatment of the acute and subacute forms

I think that, as the very first element in the treatment, opium should be given. It has been shown that intussusception depends upon disordered peristaltic movements in a limited segment of the bowel. Opium stills all peristaltic movements, and places the bowel in a condition of physiological rest. When the patient is under the influence of the drug the intussusception cannot well increase in size, although the process of strangulation may still progress. The drug, however, must be given with caution, and its effects closely watched. It must not be forgotten that opium may mask the principal symptoms, and may bring about so great a relief that the surgeon may be misled into believing that a permanent cure has followed.

With regard to the question of feeding, no nourishment should be given by the mouth in acute cases. At the most, the patient may have a little ice to suck. In acute cases the question of feeding does not really arise.

The next element in the treatment consists in attempting to reduce the invagination by enemata. In acute cases this measure should be adopted as soon as the patient is under the influence of opium. In a really acute case no benefit can be expected to attend the use of enemata after—as an extreme period—the second day. Forcible enemata, given at a later stage, in acute cases, have led to rupture of the bowel; and even when such an accident has not occurred, they have appeared to do little but harm. In subacute cases successful reduction by injection has followed at almost any period of the disease, even after ten, fourteen or twenty days have elapsed. With every day that passes, however, the chances of such reduction very rapidly diminish. Pure water should be used at a temperature of 99° F.

No rules can be given to determine the amount of force to be employed. The more recent the case, the more considerable may it be. In subacute cases, the degree of pressure employed should be, at least, moderate. In any case, the injection should be retained for, at least, fifteen minutes.

Enemata of carbonic acid in these cases are, I think, to be decidedly condemned.

Failing reduction by these means, I would urge that, in acute and subacute cases, laparotomy should be performed without delay. It is the delay, and not the operation, that is so serious in these cases. Laparotomy is regarded as a last resort in these cases, whereas it should be looked upon as almost the first resort. There is no middle course open. Of certain other modes of treatment, their employment is in opposition to the chief teachings to be derived from a study of the pathology of the disease. It is true that the present mortality after laparotomy in intussusception is very high; but it can be shown distinctly that this is due to the delay in the operation, to the custom of regarding it as a last and desperate resource.

The procedure, when undertaken, should be carried out with strict antiseptic precautions. In all but exceptional cases, the incision is most conveniently made in the middle line below the umbilicus. The whole area of the abdomen can be well explored through such an incision, and any form of invagination dealt with. The intussuscepted mass should be, as far as possible, exposed in the wound, and attempts at reduction made in cases where the state of the gut would encourage such attempts. Reduction of the invagination is best effected by dragging upon the entering bowel with one hand, while the intestine about the lower end of the intussusception is gently squeezed with the other. If the bowel be found to be in a viable condition after reduction, the coil may be replaced in the abdomen, and the parietal wound closed. I am strongly of opinion that a drain should be introduced into the abdominal cavity when any evidences of more than limited peritoneal inflammation exist. The principal feature of the after treatment should be the maintaining of perfect rest in the bowel—an end effected by the administration of opium, and by feeding the patient, as far as possible, by the rectum only.

The general mortality of laparotomy in intussusception is 72.7 per cent., as estimated from 33 recorded cases. In the instances, however, where the reduction was easy, the death-rate was only 30 per cent.; while, in the cases where it was difficult or impossible, the mortality was 91.3 per cent.—*London Weekly Review.*

